# The Cu, Zn Superoxide Dismutase: Not Only a Dismutase Enzyme

**DOI:** 10.3389/fphys.2016.00594

**Published:** 2016-11-29

**Authors:** Paolo Mondola, Simona Damiano, Anna Sasso, Mariarosaria Santillo

**Affiliations:** Dipartimento di Medicina Clinica e Chirurgia, Unità di Fisiologia Umana, Università degli Studi di Napoli “Federico II,”Napoli, Italy

**Keywords:** CuZn superoxide dismutase, oxidative stress, muscarinic M1 receptor, transductional mechanisms, secretion

## Abstract

The Cu,Zn superoxide dismutase (SOD1) is an ubiquitary cytosolic dimeric carbohydrate free molecule, belonging to a family of isoenzymes involved in the scavenger of superoxide anions. This effect certainly represents the main and well known function ascribed to this enzyme. Here we highlight new aspects of SOD1 physiology that point out some inedited effects of this enzyme in addition to the canonic role of oxygen radical enzymatic dismutation. In the last two decades our research group produced many data obtained in *in vitro* studies performed in many cellular lines, mainly neuroblastoma SK-N-BE cells, indicating that this enzyme is secreted either constitutively or after depolarization induced by high extracellular K^+^ concentration. In addition, we gave many experimental evidences showing that SOD1 is able to stimulate, through muscarinic M1 receptor, pathways involving ERK1/2, and AKT activation. These effects are accompanied with an intracellular calcium increase. In the last part of this review we describe researches that link deficient extracellular secretion of mutant SOD1^G93A^ to its intracellular accumulation and toxicity in NSC-34 cells. Alternatively, SOD1^G93A^ toxicity has been attributed to a decrease of K_m_ for H_2_O_2_ with consequent OH radical formation. Interestingly, this last inedited effect of SOD1^G93A^ could represent a gain of function that could be involved in the pathogenesis of familial Amyotrophic Lateral Sclerosis (fALS).

## Introduction

Superoxide dismutases (SODs) belong to the family of isoenzymes involved in the scavenging of O_2_ radicals. All mammalian cells possess three isoforms of superoxide dismutase enzymes; the cytosolic copper-zinc dimeric form, known as SOD1, the mitochondrial tetrameric manganese superoxide dismutase or SOD2 (Weisiger and Fridovich, 1973) and the extracellular tetrameric Cu,Zn superoxide dismutase or SOD3 (Marklund, 1982). All these enzymes catalyze the same reaction converting the oxygen radical in molecular oxygen and hydrogen peroxide H_2_O_2_ through the alternate reduction and reoxidation of Cu^2+^ for SOD1 and SOD3 and Mn for SOD2; the H_2_O_2_ is then enzymatically converted by catalase and glutathione peroxidase in molecular oxygen and H_2_O.

The discovery of 32 kDa homodimeric protein Cu,Zn superoxide dismutase, SOD1, is due to McCord and Fridovich studies (McCord and Fridovich, 1969). The SODs and the recognition of their distribution in various aerobic organisms have important implications: first, the production of the superoxide radical is inevitable because it is closely related to the metabolism of molecular oxygen in mitochondria and in cellular membranes (Damiano et al., 2013, 2015; Accetta et al., 2016); moreover, high levels of this radical, hydrogen peroxide, and hydroxyl radical, reactive oxygen species (ROS) cause oxidative stress and high cell toxicity because they react with many organic molecules. The term “oxidative stress” is referred to an imbalance between high levels of ROS and low cellular antioxidant defenses (Halliwell, 2014). The SODs represent the first enzymatic defense system against radical damage by oxygen: thus, this enzyme is essential for all aerobic organisms, but not for anaerobes. In support of this hypothesis, McCord believed that the existence of an aerobic organism depends mainly on its ability to produce SODs since its deficiency is responsible for oxygen sensitivity and allows survival only in an anaerobic environment.

The SOD1 is ubiquitous in mammalian cells and is expressed at relatively high levels also in blood vessels; in normal mouse aorta the activity of SOD1 accounts for 50–80% of total SOD activity; a similar pattern of expression was observed in human arteries (Horiuchi et al., 2004; Santillo et al., 2015). In physiological conditions, the superoxide dismutases, together with the non-enzymatic ROS scavengers as vitamins E, A, and C maintain a steady state between oxidant and antioxidant systems (Russo et al., 2011). The dysregulation in redox homeostasis, determined by an imbalance between ROS production and scavenging capacity, determines considerable cellular damage as membrane lipoperoxidation, nucleic acid and structural alterations of proteins contributing to neurodegenerative and cardiovascular diseases.

The purpose of this review is to highlight new SOD1 functions in addition to its canonic role of oxygen radical enzymatic dismutation.

In the last years, many data obtained in *in vitro* studies performed in many cellular lines, mainly neuroblastoma SK-N-BE cells, indicate that SOD1 is secreted and is able to activate, through muscarinic M1 receptor, cellular pathways involving ERK1/2 and AKT activation; these effects are associated with intracellular calcium increase that is further accentuated when these cells are stimulated with mutated SOD1^G93A^.

## Cellular localization of SOD1 and evidences for constitutive SOD1 secretion

SOD1 is highly present in the cytosol but is also partially localized in the mitochondrial matrix (Fukai and Ushio-Fukai, 2011) where, instead, SOD2 is particularly expressed. The intracellular cytosolic SOD1 localization has been a matter of debate; recent evidences, performed in transfected mouse neuroblastoma neuro2 cells, demonstrated that both wild type SOD1 (wt-SOD1) and SOD1 mutants are distributed into luminal structures of endoplasmic and Golgi apparatus (Urushitani et al., 2008).

The first experimental evidence that some cellular lines could be able to secrete the Cu,Zn superoxide dismutase date back to many years ago when we, for the first time, showed the secretion of this protein by experiments performed in hepatocytes and fibroblasts (Mondola et al., 1996), neuroblastoma SK-N-BE cells (Mondola et al., 1998; Gomes et al., 2007; Polazzi et al., 2013) and in thymus derived epithelial cells (Cimini et al., 2002). Interestingly, in further studies we observed the noticeable presence of SOD1 in human serum lipoproteins, mainly in low density (LDL) and high density (HDL) lipoproteins, ascribing to this protein a protective role against the lipoperoxidation (Mondola et al., 2000). In addition, we demonstrated that in human neuroblastoma SK-N-BE cells, that show a greater sensitivity to glucose deprivation-induced cytotoxicity due to enhanced sensitivity to ROS (Shutt et al., 2010), SOD1 export takes place in normal conditions and is increased following oxidative stress (Mondola et al., 1996, 1998).

Successively, we showed that SOD1 in human neuroblastoma SK-N-BE cells is exported through a microvesicular secretory pathway that is impaired by brefeldin-A (BFA), and by 2-deoxyglucose, and sodium azide, which reduces ATP intracellular pool (Mondola et al., 2003). Moreover, in further studies we indicated that in SK-N-BE cells SOD1 was able to activate PLC-PKC pathway increasing intracellular calcium concentration (Mondola et al., 2004).

## Inducible SOD1 secretion in excitable cells

Another important aspect was the discovery that besides the constitutive SOD1 export, the secretion of this enzyme is also induced. To this respect, we showed (Santillo et al., 2007) that SOD1 is actively released from rat brain synaptosomes as well as from rat pituitary GH3 cells that represent a good model to study the inducible SOD1 release since they possess all the neuronal protein machinery involved in synaptic vesicle exocytosis. In these cellular models we demonstrated, by confocal images and Western Blotting experiments, that the depolarization, induced by high extracellular K^+^ concentration, induced SOD1 release correlated with an increase of intracellular calcium influx; these effects were abolished by removal of extracellular calcium as well as by cell preincubation either with calcium chelator EGTA or with Botulinum toxin A that cleaves the SNARE protein, SNAP-25. In addition, in the attempt to evaluate the possible role carried out by SOD1 export, we recently demonstrated, in SK-N-BE neuroblastoma cell line, that this enzyme is able, through the involvement of muscarinic M1 receptor, to activate ERK1/2 and AKT in a dose and time-dependent manner. This effect was remarkably reduced by M1 receptor silencing as well as by using M1 antagonist pirenzepine (Damiano et al., 2013).

## Pathway of SOD1 export

The majority of the members of growth factors, Fibroblast growth factor 1 and 2 (FGF-1 and FGF-2), are exported by Endoplasmic Reticulum-Golgi (ERG) dependent secretory transport. However, FGF-1 and the 18 kDa isoform of FGF-2 have been shown to be secreted by an alternative pathway being directly translocated from the cytoplasm into the extracellular space. Analogously, also interleukin 1β (IL-1β) has been reported to be secreted by a vesicular non-classical export pathway.

Soluble proteins classically contain N-terminal signal peptides that direct them to the translocation apparatus of the Endoplasmic Reticulum (ER) (Walter et al., 1984). Following vesicular transport from the ERG to the cell surface, proteins are then released extracellularly by fusion of Golgi-derived secretory vesicles with the cellular membrane (Palade, 1975; Rothman and Wieland, 1996; Schekman and Orci, 1996; Mellman and Warren, 2000). In addition, non-classical protein secretion is both energy and temperature dependent and can be stimulated or inhibited by various treatments (Cleves, 1997; Hughes, 1999). The list of proteins that could be exported from cells in the absence of a functional ERG system (unconventional secretory pathway), as IL-1β and galectin-1 (also referred to as L-14), is continually growing; for further data see the review of Nickel (2003). It is known that SOD1 is mainly a cytosolic protein that does not possess a signal peptide for translocation into inner membrane of ER. For this reason SOD1 secretion should bypass the canonical ERG secretory pathway. We previously demonstrated that BFA as well as 2-deoxyglucose and sodium azide (NaN_3_), impairs SOD1 export (Mondola et al., 2003). In our opinion, the treatment with BFA probably dysregulates not only the classical secretory ERG pathway but also the microvesicular membrane traffic of unconventional protein secretion or alternative protein export. However, the export pathway of SOD1 has not yet been clarified since some authors (Urushitani et al., 2008) indicated an involvement of the ERG pathway; conversely, further studies performed by confocal microscopy and immunoblotting (Atkin et al., 2014) showed that wt-SOD1 or SOD1^A4V^, another mutant form of SOD1, were not detected in the ER in NSC-34 cells. Furthermore, these authors showed that SOD1^A4V^ inhibits secretory protein transport from the ER to the Golgi apparatus.

## Impaired SOD1 secretion in amyotrophic lateral sclerosis

Amyotrophic lateral sclerosis (ALS) is an adult onset, neurodegenerative disease characterized by selective death of the upper and lower motor neurons of the brain and spinal cord. Symptoms include muscle atrophy, spasticity, paralysis and eventual death from respiratory failure within 3–5 years of diagnosis. While ALS mostly affects patients without family histories of the disease, 5–10% of ALS is familial (fALS).

Clinical and pathological processes indicate that ER stress represents a key pathway involved in cell death. In the transgenic SOD1^G93A^ ALS rat model unfolded protein response and ER stress-induced apoptosis has been observed (Atkin et al., 2006); an unfolded protein response, including induction of stress sensor kinases, chaperones, and apoptotic mediators, has been shown also in spinal cord motor neurons of human patients with the sporadic form of ALS (sALS) that is not restricted to SOD1 mutations (Atkin et al., 2008).

However, the link between the pathogenic mechanisms of fALS and sALS remains unclear, since in the latter, misfolded wt-SOD1 protein activates the same neurotoxic mechanism that is invoked by SOD1 mutants in fALS (Bosco et al., 2010; Forsberg et al., 2010).

SOD1 and other proteins are misfolded in fALS and in sALS, but it is not clear how this triggers ER stress, fragmentation of the Golgi apparatus, disruption of axonal transport and apoptosis. Nearly 20% of fALS is caused by SOD1 gene mutations (Neumann et al., 2006). Indeed, the majority of SOD1 mutants maintain their enzymatic activity suggesting the occurrence of gain of toxic activity function rather than a simple loss of function (Strong et al., 2005; Dion et al., 2009).

Turner et al. (2005) demonstrated an impaired constitutive extracellular secretion of mutant SOD1 in NSC-34 cells that induces frequent cytoplasmic inclusions and protein insolubility. These data link the deficient secretion of mutant SOD1 with intracellular protein aggregates and toxicity in NSC-34 cells. In addition, these authors showed that in a transgenic rat model of ALS the chronic intraspinal infusion of exogenous human wt-SOD1 significantly delayed disease progression suggesting a novel extracellular role for SOD1 in ALS; therefore extracellular delivery of human wt-SOD1 could improve clinical disease in transgenic ALS rats supporting a novel extracellular role for mutant and wt-SOD1 in ALS pathogenesis and therapy, respectively.

Moreover, recent studies demonstrate that SOD1 mutants can also be exported; in fact, although SOD1 inclusions are exclusively intracellular, there is evidence that these inclusions can escape from the cytoplasm and come into contact with nearby neuronal cells (Münch et al., 2011). In addition, in transgenic mice, carrying SOD1 mutations, toxic effects to motor neurons by microglia activation were observed (Urushitani et al., 2006; Zhao et al., 2010).

The microglia cells can carry out an important role in ALS progression (Pramatarova et al., 2001) since microglia activation can be observed before neuron loss in transgenic mice expressing human SOD1 mutants (Alexianu et al., 2001). Data from experiments conducted in wt/mutant SOD1 chimeric mice demonstrated that mice with wt-SOD1 glial cells live longer, suggesting that glial cells containing SOD1 mutants contribute to disease (Clement et al., 2003). The pathogenic role of mutated SOD1 has not yet been completely clarified. The involvement of deranged inflammatory processes in ALS pathogenesis has been largely suggested.

It is believed that ER stress is triggered when proteins accumulate within the ER lumen. ER stress can also be caused by other mechanisms such as inhibition of ERG transport, which results in accumulation of secretory proteins within the ER (Høyer-Hansen and Jäättelä, 2007; Preston et al., 2009; Araki et al., 2012). Our first demonstrations of SOD1 secretion in many cellular lines stimulated many researches in order to study the secretory pathway of this protein in a ALS.

As it is known, mutations of SOD1 in transgenic rats or mice induced motor neuron disease (Gurney, 1994; Howland et al., 2002) and are associated with about 20% of fALS. In addition, SOD1^G93A^ causes protein aggregation inducing cellular damage (Bendotti and Carrì, 2004; Bruijn et al., 2004). As has been previously evaluated, about one third of all proteins transit trough the ER and Golgi compartments (Ghaemmaghami et al., 2003) and the coat protein II complex, (COPII) constituted by five subunits: Sar1, Sec23, Sec24, Sec13, and Sec31 (Lee and Goldberg, 2010) is considered to carry out a central role in this process. Restoration of ERG transport by over-expression of coatomer coat protein II subunit Sar1 protected against inclusion formation and apoptosis, linking dysfunction in ERG transport to cellular pathology. These results suggest that several cellular events in ALS are linked into a single mechanism occurring early in mutated SOD1 expressing cells. Therefore, even if mutated SOD1 inhibits ERG transport, the mechanism by which SOD1 mutants are toxic is still unknown. The microglial activation, due to mutated SOD escaped from the cell could have, a relevant role. In a recent review, it is suggested that post-translational modifications of SOD1 could cause wild type to adopt a “toxic conformation” that is similar to fALS-linked SOD1 variants. These observations, together with the detection of misfolded wt-SOD1 within human post mortem sALS samples, have been used to support the controversial hypothesis that misfolded forms of wt-SOD1 contribute to sALS pathogenesis (Rotunno and Bosco, 2013).

## SOD1 as neuromodulator

Many studies pointed out that muscarinic receptors are widely expressed in the central nervous system controlling several neuronal functions; in addition, it has been shown that the activation of muscarinic M1 acetylcholine receptor coupled to G_q11_ increases extracellular signaling-regulated protein kinase (ERK 1/2) modulating synaptic transmission (Hamilton et al., 2001; Anagnostaras et al., 2003; Scheiderer et al., 2006; McCoy and McMahon, 2007). In further studies, we demonstrated (Mondola et al., 2004) that the incubation of SOD1 with SK-N-BE cells activated a PLC–PKC-dependent pathway inducing a relevant intracellular [Ca^2+^]_i_ increase. The specificity of SOD1 surface binding to SK-N-BE membrane was strengthened by the fact that neither biotinylated human thyroglobulin nor human thyroperoxidase bound to the neuroblastoma cell membranes. Moreover, our data showed that the effect of SOD1 was independent of dismutase activity, since the SOD mimetic substance, MnTMPyP, failed to produce the same effects. On the other hand, ApoSOD (free metal type 1 superoxide dismutase) was able to reproduce the same SOD1 effects mentioned above suggesting a new function of superoxide dismutase independently from its dismutase activity. Notably, SOD1 induced an increase in [Ca^2+^]_i_ by intracellular and extracellular dependent mechanisms. Indeed, since the increase in [Ca^2+^]_i_ was only partially reduced in the free calcium medium, we hypothesized that intracellular calcium stores were involved in this change. In addition, the ability of 0.1 μM ω-conotoxin and 1 μM nimodipine to inhibit only in part this effect indicated that N and L types of VGCCs could elicit SOD1-dependent intracellular Ca^2+^ increase; therefore this effect should be better investigated. Moreover, our data indicate that U73122, the PLC inhibitor, completely reduced SOD1-induced [Ca^2+^]_i_ increase (Mondola et al., 2004). These and previous data pointed out that SOD1, through an increase of calcium-dependent signaling, could affect different biological functions besides acting as scavenger of superoxide radical. The paracrine role carried out by SOD1 could also explain the other function ascribed to this protein (Mondola et al., 2002; De Felice et al., 2004; Viggiano et al., 2012; Terrazzano et al., 2014).

## SOD1 muscarinic M1 receptor interaction

Muscarinic M1 receptor represents the predominant subtype of muscarinic acetylcholine receptors in the central nervous system (CNS); this receptor, localized at post-synaptic level in the cortex, hippocampus, striatum, and thalamus (Ellis, 2002), activates the extracellular signal-regulated kinase pathway (Berkeley et al., 2001). Recently, it has been demonstrated that the stimulation of M1 receptor attenuates the disease progression in subjects affected by Alzheimer disease (AD) (Caccamo et al., 2006); moreover, it has been hypothesized that selective activation of muscarinic M1 receptor could lead to improvement of cognitive aspects in schizophrenic patients.

Our previous data showed the secretion of SOD1 in many cellular lines (Mondola et al., 1996, 1998, 2002); in addition, further cytofluorimetric studies demonstrated a specific SOD1 interaction with the plasmamembrane of neuroblastoma SK-N-BE cells that results in an activation of phospholipase C (PLC) transductional mechanisms associated to a slight increase of intracellular calcium concentration (Mondola et al., 2004; Damiano et al., 2013).

Using rat pituitary GH3 cells that express the neural protein machinery involved in vesicle neurosecretion, we demonstrated the presence of SOD1 in large core dense vesicles; moreover, we showed that SOD1 is secreted in response to high (55 mM) extracellular K^+^ concentration (Santillo et al., 2007).

Recently, we showed that SOD1 activates muscarinic M1 receptor inducing a phosphorylation of ERK1/2 and AKT in a dose and time dependent manner in neuroblastoma SK-N-BE cells; in fact, in presence of M1 antagonist pirenzepine, ERK1/2 and AKT phosphorylation, induced by SOD1, was strongly prevented. A further involvement of M1 muscarinic receptor was confirmed by knocking-down experiments using two sequences of human M1 receptor small interfering RNAs and by a M1 stimulator (oxotremorine) that respectively decrease and increase activation of ERK1/2 and AKT, suggesting a neuromodulatory effect of SOD1 (Damiano et al., 2013).

## Conclusions

This review is an update and expansion of many of our researches dating back to many years ago when we, for the first time, gave experimental evidences of SOD1 secretion in different cellular lines. Based on the evidence of secretory pathway dysfunction in ALS, Turner et al. (2005) confirmed our previous researches related to SOD1 secretion also in mouse motoneuron like NSC-34 cells; in addition, these authors linked deficient extracellular accumulation of SOD1 mutants to intracellular aggregates and toxicity in NSC-34 cells demonstrating that extracellular delivery of human wt-SOD1 improved clinical disease in transgenic ALS rats.

Previous research (Yim et al., 1996) shows that, in transgenic mice, both the wt-SOD1 and the SOD1^G93A^ exhibit identical catalytic activity for the dismutation of superoxide anion, while the SOD1^G93A^ shows a decrease of Km for H_2_O_2_ with a consequent OH° radical formation. Therefore, ALS symptoms observed in SOD1^G93A^ transgenic mice could be caused by a gain-of-function of the mutated form of the enzyme.

This effect can be due to SOD1^G93A^ fALS mutant that exhibits a higher activity for catalyzing the generation of free radicals at subsaturating level of H_2_O_2_. In addition, this enhancement of the free radical-generating function will also promote inactivation of the mutant enzyme which may lead to the release of copper ions from the inactivated protein that may be in part, responsible for the development of ALS symptoms. However, the nature of this cytotoxic gain-of-function of the fALS mutant is yet to be identified.

Another more recent research pointed out that wt-SOD1 was present in the cytoplasm and in less extent in the nuclei of motor neurons, whereas SOD1^G93A^ mutant was strongly localized in the nucleus (Gertz et al., 2012). wt-SOD1 has also been detected in peroxisomes of rat hepatocytes and fibroblasts (Dhaunsi et al., 1992; Wanders and Denis, 1992). The proteasome activity analysis, responsible for misfolded protein clearance, performed in the two subcellular compartments, demonstrated proteasome impairment only in the cytoplasm. The effect of SOD1^G93A^ exclusion from nuclei, analyzed after oxidative stress, showed a higher DNA damage compared with those expressing wt-SOD1, possibly because of a loss of nuclear protection. The toxicity of SOD1 mutants could be attributed to an initial misfolding (gain of function) reducing nuclear protection from the active enzyme (loss of function in the nuclei), a process that may be involved in ALS pathogenesis (Sau et al., 2007).

**Figure 1:**
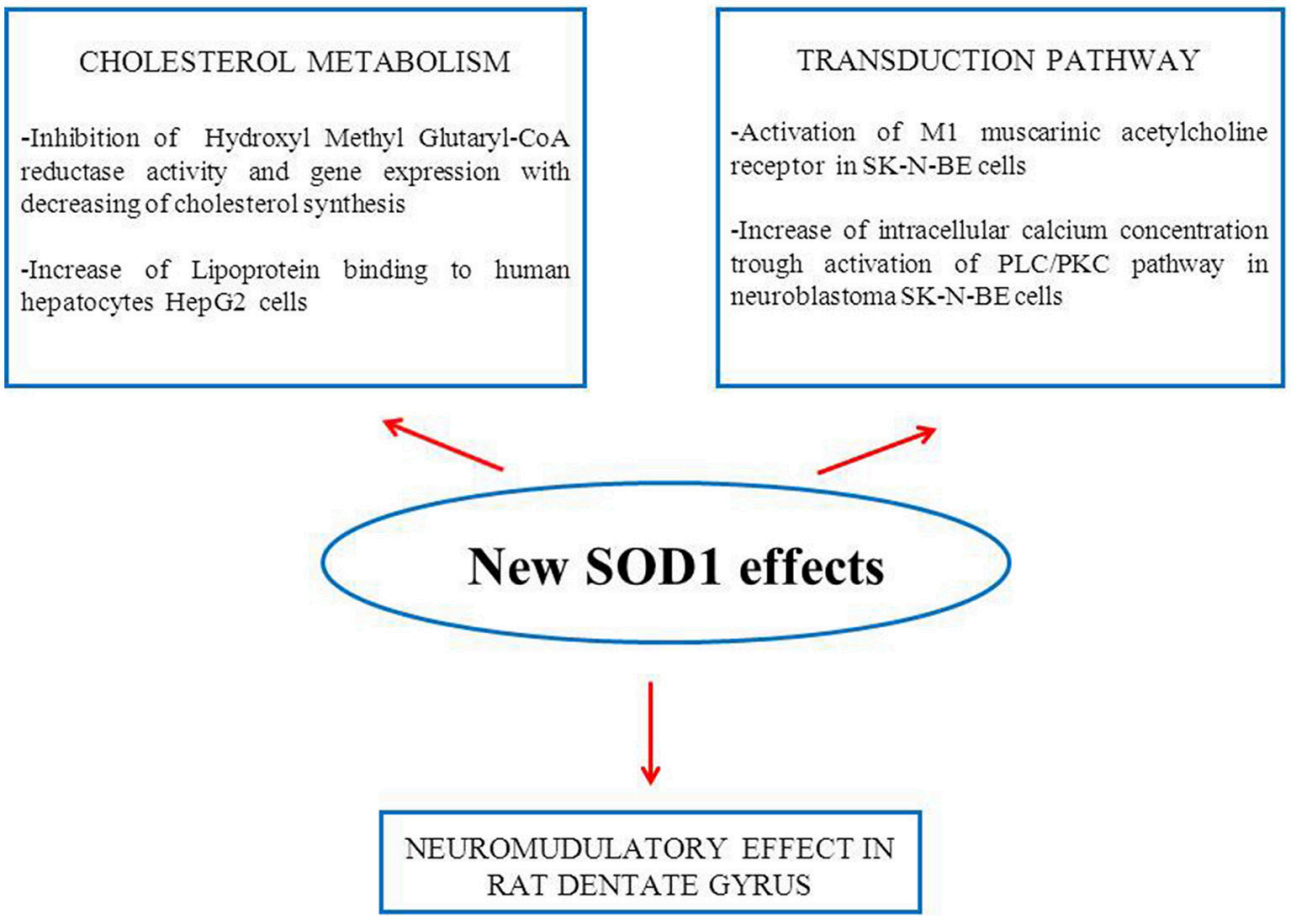
**Schematic representation of new SOD1 effects obtained in ***in vitro*** and ***in vivo*** experiments**.

**Figure 2:**
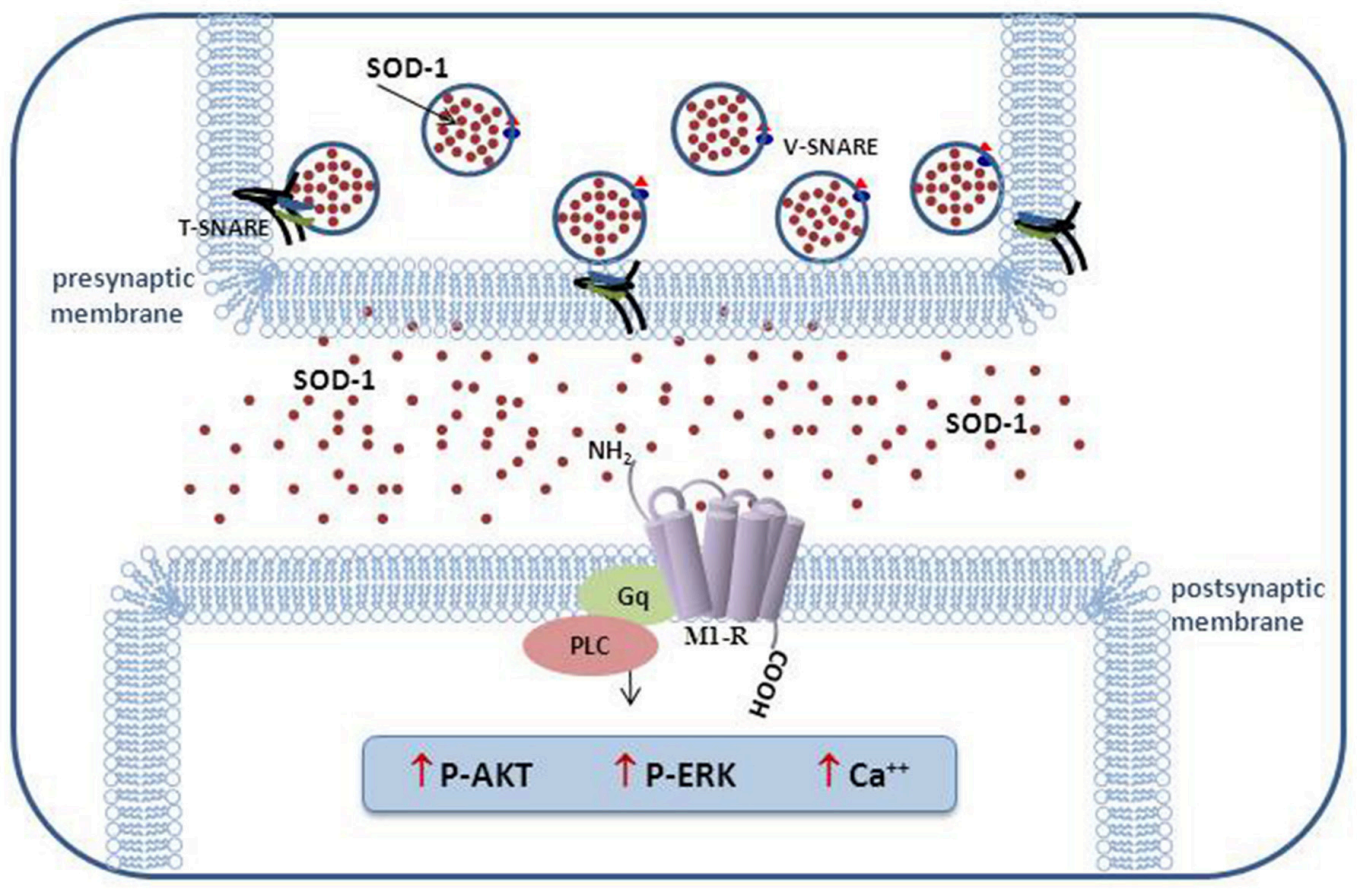
**Schematic representation of vesicular SOD1 secretion and transduction pathways activated in excitable cells**.

The results of our research performed in the last decade indicate that this enzyme cannot be considered exclusively as an antioxidant molecule able to carry out the dismutation of superoxide anion. In Figure 1, in addition to the activation of the transduction pathway involving M1 muscarinic receptor, other inedited SOD1 effects on cholesterol metabolism and synaptic modulation in rat dentate gyrus are shown (Mondola et al., 2002; De Felice et al., 2004; Viggiano et al., 2012). The inducible SOD1 vesicular mediated secretion and activation of ERK1/2 and AKT through SOD1-M1 muscarinic receptor pathway, associated to increased intracellular calcium concentration, indicates a paracrine role of this molecule as shown in Figure 2 (Damiano et al., 2013). The mechanism by which mutated SOD1^G93A^ causes ER stress and Golgi apparatus fragmentation, in addition to accumulation of misfolded or unfolded proteins at the ER lumen, has been widely studied but has not yet been completely clarified (Turner et al., 2005; Turner and Atkin, 2006; Nassif et al., 2010). Moreover, in further studies, it could be worthwhile to evaluate if the mutated SOD1^G93A^, analogously to wt-SOD1, induces the same effect on the activation of muscarinic M1 receptor involved in ERK/AKT/${\text{Ca}}_{\text{i}}^{++}$ pathway modulation.

## Author contributions

PM Conceived and wrote the review article. SD, AS, and MS contributed to write the paper.

### Conflict of interest statement

The authors declare that the research was conducted in the absence of any commercial or financial relationships that could be construed as a potential conflict of interest.
